# Bisacodyl removal from contaminated solution by synthesized mesoporous silica using experimental design method

**DOI:** 10.1186/s13568-018-0673-5

**Published:** 2018-09-10

**Authors:** Mohammad Hossien Salmani, Mohammad Hassan Ehrampoush, Mehdi Mokhtari, Bahar Eftekhar

**Affiliations:** 1 Department of Environmental Health Engineering, School of Public Health, Faculty of Public Health, Shahid Sadoughi University of Medical Sciences, Yazd, Islamic Republic of Iran; 2 Environmental Sciences & Technology Research Center, Department of Environmental Health Engineering, Shahid Sadoughi University of Medical Science, Yazd, Islamic Republic of Iran

**Keywords:** Mesoporous silica, Bisacodyl, Removal, DOE

## Abstract

Most of the medicinal compounds are entered no change into municipal wastewater and more than 90% of it remains in the wastewater. Mesoporous silica is known as thermally stable materials with controllable porosity and morphology. It is specified that these materials possess external and internal surfaces that can be selectively adsorbed the various compounds. In the present study, the synthesized mesoporous silica was studied to remove bisacodyl from polluted solutions. Mesoporous silica was synthesized by simple chemical method from tetraethylorthosilicate, ethanol (C_2_H_5_OH) and deionized water at 70 °C temperature. Characteristic of synthesized mesoporous silica was investigated by scanning electron microscopy. The effective parameters such as contact time, pH, adsorbate dose, and initial bisacodyl concentration were optimized for bisacodyl removal using 27 batch adsorption experiments according to design of experiment. The residual bisacodyl concentration was measured by UV–Vis spectrometer at the maximum wavelength of 580 nm. The statistical test and linear regression model were used by MINITAB 16 software for investigation of the main and interaction effects of each factor in the adsorption process. The ANOVA result showed 84% of bisacodyl was removed by synthesized mesoporous silica at optimum condition. The result of regression (R^2^ > 0.99) indicated that the adsorption process followed the Langmuir isotherm and second order kinetic at optimum conditions. The mesoporous silica is an efficient adsorbent for removing of bisacodyl from the polluted solutions so that it can be used for refining wastewaters containing medical compounds such as bisacodyl.

## Introduction

Water is a suitable solvent for many compounds therefore, water resources are always at risk of contamination (Crittenden et al. 2012). The residual drug is one of the organic pollutants for water resources. Nowadays, due to risk potential of water resources, excessive consumption of drugs is considered as a major concern of chemical environment management (Heberer 2002; Daughton and Ternes 1999). After consumption of a drug, it is excreted of urine and feces completely (non-decomposed) or in metabolized form. Unlike other pollutants, because of the importance of health and economy, control of drug pollutants is necessary for the environment. According to the chemical structure of pharmaceutical compounds, the common methods of water purification are not often effective in drug removal from the polluted waters (Nair Abhilash 2012). Therefore, many pharmaceutical compounds are not removed in conventional wastewater treatment (Heberer 2002) and more than 90% of residual drug remain, completely. So, a remarkable amount of pharmaceutical compounds gets into surface water and groundwater (Ternes 1998).

Residual of drugs get into the environment in two ways: the first way is largely unintentional and unavoidable which it inters into the environment via urine, feces, and bathing. The second way is to discharge the solid medical waste, domestic waste, and drug transmission into the environment (Daughton 2008). Specific effects of drugs on environment and ecosystems are not completely known and have recently become the topic of interest for researchers. Also, the controller rules for these pollutants are not completely enforced yet and researchers are interested in the field of drug removal study from polluted water (Nair Abhilash 2012). Drugs include a wide range of chemicals and are divided into several groups (Ternes 1998). One of these groups is gastrointestinal drugs that have different types. Bisacodyl, one of them, is among the class of diphenyl-methane which is used as a laxative drug for short-term treatment of constipation, the evacuation of the colon before radiological examination of the abdomen, endoscopy and before or after surgery (Ali 1979; Deepali 2011). Table 1 shows the physical and chemical properties of bisacodyl. Partially or fully hydrolysis of bisacodyl produced the decomposition of one or two groups of acetyl structure of bisacodyl that these are considered as pollutant compounds (Ali 1979).

**Table 1:** Physical and chemical properties of bisacodyl

Parameters	Bisacodyl
Formula	C_22_H_19_NO_4_
Molecular weight	361.397 g/mol
Structure	
Log k_ow_	3.37
Biological half-time	16 h

Recently, many techniques have been used to remove contaminants from the polluted water and wastewater. The adsorption processes more than other techniques had been developed and applied to remove the various pollutants from aqueous solutions (Iram et al. 2010; Shen et al. 2011; Kakavandi et al. 2014). Porous silica have cavities in size of 2–50 nm, are known by the general name of mesoporous compounds, have a unique performance in adsorption (Anbia and Mohammadi 2009). Uniform nanometer pores of mesoporous silica materials make it possible to separate molecules based on size difference. One of the important purposes of chemical engineering is to synthesize and design a suitable adsorbent that have the ability to adsorb pharmaceutical compounds, high adsorption capacity, as well as sufficient and adequate stability in a pharmaceutical environment. Therefore, the purpose of this study was to synthesize silica mesoporous by the chemical method to achieve the maximum removal by optimization of effective parameters on bisacodyl removal from aqueous solution for treatment of polluted waters.

## Materials and methods

The experimental study was conducted in the batch system at the laboratory scale. The 27 experiments were designed to investigate the effects of adsorbent mass, pH and the initial concentration on the removal efficiency of mesoporous by design of experiment. In the present study, ethanol and bisacodyl (C_22_H_19_NO_4_) were purchased by the Iran Sobhan Company. Cetyl trimethyl ammonium bromide, tetra ethyl ortho silicate, iron (III) chloride, sodium acetate, acetic acid, sodium hydrogen phosphate, potassium dihydrogen phosphate, phenanthroline and ammonia were purchased by Merck Company. The 0.05 N acetate buffer solution was used to fix the pH of the solution in 4, and for pH of 7 and 9 used the 0.05 N phosphate buffer solution. Absorption of samples was taken by Shimadzu (UV-1800) UV–Vis spectrometer and the solution pH was measured by Hanna pH meter that was firstly calibrated with buffer solutions. In all experiments, an orbital shaker was used to mix the suspension for suitable contact of adsorbent and bisacodyl at 220 RPM. Erlenmeyer flask was closed with cap to prevent pouring out of solution during shaking.

### The synthesis of mesoporous silica

To synthesize mesoporous silica (MCM-41), 50 g of CTAB (Cetyl trimethyl ammonium bromide) was dissolved in 100 ml of 50% v/v water- ethanol and then 50 ml of sodium acetate were added to adjust the pH 12. The mixture was stirred at the 30 °C in a shaker-incubator (220 RPM) for 10 min. Then 25 ml of TEOS (tetra-ethyl ortho silicate) was added to the mixture and the resulting gel was aged at 100 °C for 24 h. For obtaining gel, the molar ratio of compounds were TEOS = 1, CTAB = 0.22, NH_3_ = 11, sodium acetate = 0.034, water = 155, ethanol = 1 (Teymouri et al. 2011). The white precipitate was separated by 0.45 μ filter paper, washed with deionized water and dried at the 90 °C for 1 h. Finally, the obtained precipitate was calcined in the electrical furnace at the 550 °C for 2 h.

### Adsorption experiments

A stock solution was prepared by dissolving 5 mg of bisacodyl in 100 ml of ethanol. The needed initial concentrations for working and standard solutions were prepared using the stock solution in 50 ml by adding 0.05 N acetate buffer solution to achieve a specific volume. The effect of contact time on removal efficiency was optimized by 6 levels of 1, 3, 9, 12, 18, and 24 h in initial pH of 6.5, a concentration of 5.0 mg/l, and the adsorbent dose of 0.3 g. In order to decrease the number of experiments, an experimental design method was used to design of experimental condition (Table 1). Adsorption experiments were designed at three levels of adsorbent dose (0.3, 0.5 and 0.7 mg/100 ml), three levels of initial bisacodyl concentration of (5, 25, and 50 mg/l) and three levels of pH of (4, 7, and 9) that are presented in Table 2. Then, based on designing tests with varying of pH, adsorbent dose, and initial concentration of bisacodyl, the 27 batch adsorption experiments were performed to investigate bisacodyl removal by the synthesized mesoporous silica, separately.

**Table 2:** The amounts and levels of the studied parameters

Factors	Level 1	Level 2	Level 3
pH	4	7	9
Initial concentration (C_0_)	5	25	50
Adsorbent dose (m)	0.3	0.5	0.7

The adsorbent mass of 0.5 mg in 100 ml, bisacodyl initial concentration of 5, 25, 50 mg/l, contact time of 18 h and pH of 7 were used to determine adsorption isotherm and bisacodyl initial concentration of 5 mg/l, pH of 7, adsorbent mass of 0.5 mg in 100 ml and contact time of 1, 3, 6, 9, 12, and 18 h were used to determine adsorption kinetic.

### Measurement

After removal process, adsorbent particles were separated from the suspension by 0.45 μ followed by centrifuge and then the remaining concentration of bisacodyl was measured in the clear filtrated solution. For this purpose, the volume of 2, 0.4 and 0.2 ml of samples were respectively taken for different concentrations of 5, 25 and 50 mg/l poured into a container and were added 1 ml of 2 M iron (III) chloride. The mixtures were agitated on the shaker at the 70 °C for 15 min. After cooling, 5 ml of 1–10 phenanthroline reagent was added and diluted to achieve the volume of 25 ml with distilled water. Absorption of the solution in standard and unknown samples was measured by UV–Vis spectrometer at the wavelength of 580 nm.

## Results

**Fig. 1:**
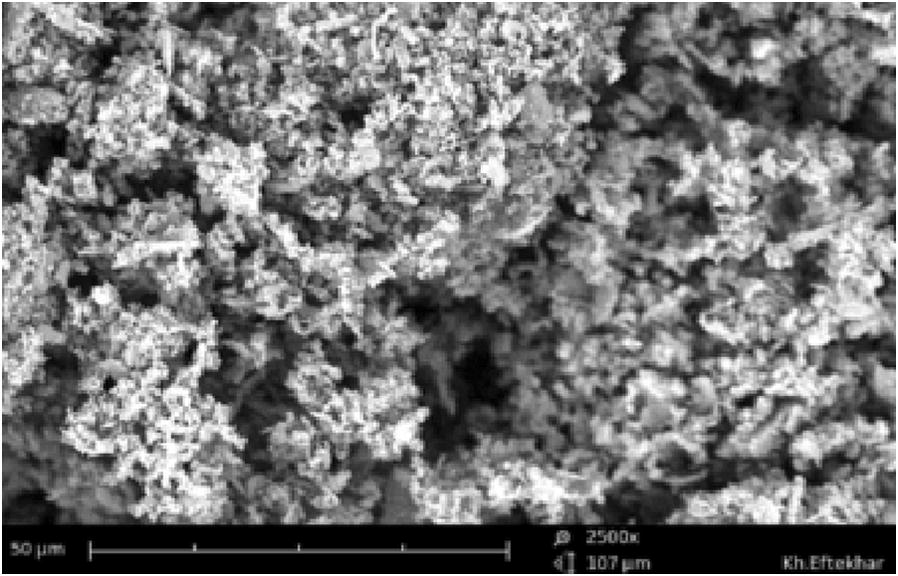
The SEM image of mesoporous silica sample with magnification of ×2500

Scanning electron microscope (SEM) was used for checking out the structure and size of nanoparticles. The SEM image of synthesized mesoporous silica is shown in Fig. 1.

### Optimization of main parameters on removal process

The batch adsorption process depends on various factors such as pH, temperature, adsorbent dose, the concentration of pollutant, adsorbent kind, and agitation time. The present study, the effect of contact time on removal of bisacodyl by the synthesized mesoporous silica was optimized and then the factors of initial bisacodyl concentration, adsorbent dose and pH were screened according to DOE method.

### The effect of contact time on bisacodyl removal

**Fig. 2:**
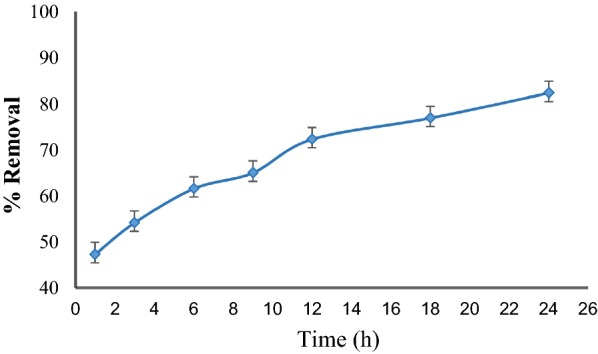
Effect of contact time on the bisacodyl adsorption process, the conditions were pH of 7, initial concentration of 5 mg/l and adsorbate mass of 0.5 mg in 100 ml. The experiments were performed in triplicate, and the mean and error bar are presented on it

The effect of contact time on bisacodyl removal efficiency is shown in Fig. 2.

### The effect of pH, initial concentration and adsorbent mass on bisacodyl removal

The obtained results from bisacodyl adsorption experiments were analyzed by analysis of variance (ANOVA) statistical test and linear regression model using Minitab 16 software. The results of the ANOVA test are shown in Table 3.

**Table 3:** ANOVA results of bisacodyl removal efficiency

Factors	D.F	Sum of square	Mean square	F	P
pH	2	1233.41	616.70	42.50	0.000
Co	2	669.85	334.93	23.08	0.000
M	2	185.41	92.70	6.39	0.022
pH. Co	4	43.04	10.76	0.74	0.590
pH. m	4	1061.48	265.37	18.29	0.000
Co. m	4	20.37	5.09	0.35	0.837
Error	8	116.07	14.51		

**Fig. 3:**
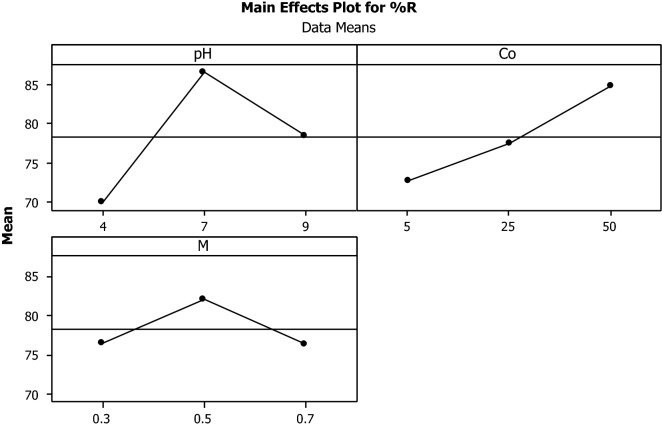
Main effects of each parameter on the bisacodyl adsorption process, initial concentration had a strong positive effect on adsorption process. The pH of 7 was the optimum for removal of bisacodyl and the adsorbent dose had a similar effect on the removal efficiency

For more clearly analyze, the main effect curves were plotted by Minitab 16 software. Figure 3 shows the main effects of each parameter in bisacodyl removal process by mesoporous silica.

**Fig. 4:**
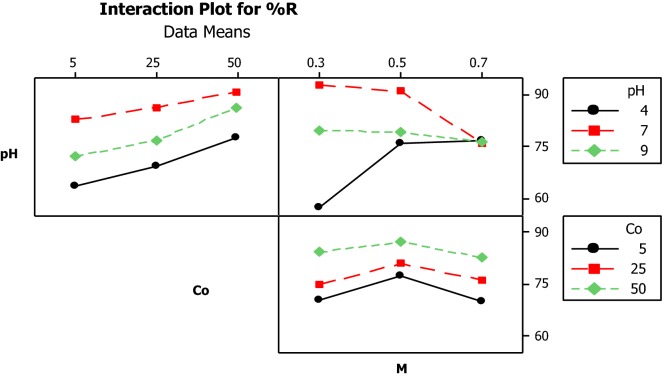
Interaction of parameter in the bisacodyl adsorption process, the lines are not parallel. It represents some evidence for effective interactions between the parameters. The significant interaction between pH and adsorbent dose observed in the bisacodyl adsorption process

Also, the interaction of parameters was studied on bisacodyl adsorption process that is shown in Fig. 4.

### Isotherm and kinetic study

The adsorption isotherm of bisacodyl removal process by the synthesized mesoporous silica was studied using Langmuir and Freundlich models. The constants of Langmuir and Freundlich isotherm model and their correlation coefficients (R^2^) are calculated and represented in Table 4.

**Table 4:** Langmuir and Freundlich constants and correlation coefficients for bisacodyl adsorption by mesoporous silica

Isotherm	Langmuir			Freundlich		
Adsorbent	q_m_	b	R^2^	n	K_F_	R^2^
Bisacodyl	7.31	0.17	0.996	0.852	2.34	0.640

**Fig. 5:**
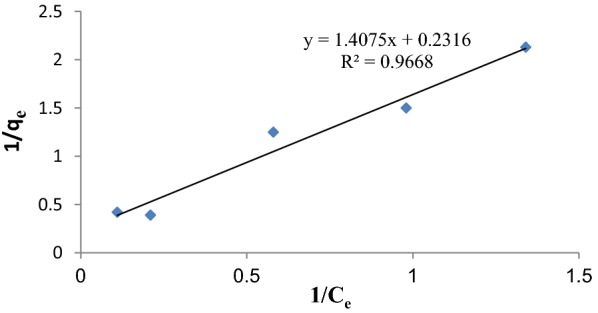
Langmuir isotherm model for bisacodyl adsorption by mesoporous silica, the experiments were performed in the initial concentration of 5, 25, 50 mg/l, contact time of 18 h and pH of 7. The results were well fitted (R^2^ = 0.967) and showed the model’s prediction for removal of bisacodyl by mesoporous silica

The linear plot of the Langmuir isotherm for bisacodyl adsorption by mesoporous silica is illustrated in Fig. 5.

**Fig. 6:**
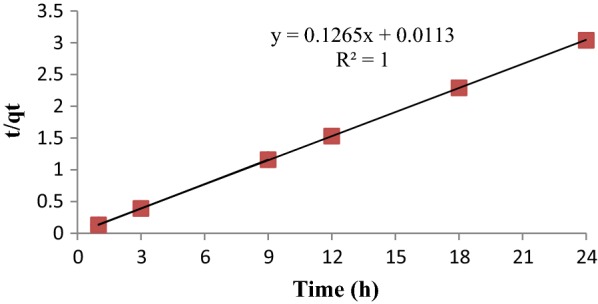
Pseudo- second order kinetic model for bisacodyl adsorption by mesoporous silica, the results were well fitted (R^2^ = 1) and showed the well agreed with the Pseudo-second order kinetic model

The experimental data of kinetic study for bisacodyl adsorption process by mesoporous silica were analyzed by the pseudo-first and second kinetic equations. Figure 6 shows a plot of the linearized form of the pseudo-first order model for the adsorption process of bisacodyl by the synthesized mesoporous silica.

## Discussion

Structure and pore size of mesoporous silica particles were checked out by SEM image. Figure 1 showed that the synthesized mesoporous exhibit the porous network. It observed the particles is the uniform in shape and have many large pores below 100 nm.

As can be seen from Fig. 2, the removal rate of bisacodyl by mesoporous silica increased with time and reached to 84% when the contact time was 24 h. The removal efficiency increased with increasing the contact time because of the opportunity for the collision, contact, and adsorption between the pollutant and adsorbent in the reaction environment. In the present study, since near 77% of bisacodyl were removed in 18 h and the adsorption process increased slowly after this time. Hence, the contact time of 18 h was considered as an optimal time which was used in next experiments. In a study conducted by Alahabadi et al. (2014) similar results were obtained. They found that the removal of the antibiotic amoxicillin from the contaminated water increased by granular activated carbon until equilibrium time (60 min) and there were no significant changes in removal efficiency after this time (Alahabadi et al. 2014).

The results of the ANOVA test (Table 3) were analyzed by means of F and P values. F-values were applied to determine the regression coefficient of factors and P-values were used to check out the significance of variables and interactions between them. In general, the larger amount of F and the lower amount of P for a term indicating the coefficient is more significant (Salmani et al. 2016). According to the Table 3, the large amount of F-value and low amount of P-value are related to the parameters of pH and initial concentration of bisacodyl, whereas the effect of adsorbent dose in adsorption process is statistically no significant than other parameters. Also, the term of pH.m is statistically more significant than other terms. By removing these terms, which were not statistically significant, the linear regression model for bisacodyl removal by the synthesized mesoporous silica adsorbent was obtained as the following equation.

1 $$ \% {\text{R }} = { 57}. 9 { } + { 4}. 1 7 {\text{ pH }} + { 6}.0 6 {\text{ C}}_{\text{o}} + { 3}. 1 1 {\text{ pH}}.{\text{m}}\quad\,{\text{F}} = 6 3 5.0 7\quad\,{\text{R}}^{ 2} = 0. 9 9 6 $$

The F and P value indicated that the form of the model chosen to explain the relationship between the factors and the removal efficiency is correct. The effective terms in the adsorption process can be well analyzed by the above equation. Equation 1 specifies that the main parameters of pH and Co have positive coefficients, in other word, these parameters have positive effects on the removal efficiency for mesoporous silica adsorbent. Also, the most effect is related to the initial bisacodyl concentration that has the highest coefficient, and pH affects the adsorption process, further. The main effect curves show deviations between high levels and low levels of each main parameter. When the effect of a factor is positive, removal efficiency increases by changing of the factor from low level to high level. In contrast to, if the effect of a factor is negative, removal efficiency will decrease when the variable is at a high level.

Figure 3 shows that initial concentration has a strong positive effect on adsorption process and therefore, by increasing the initial concentration from 5 mg/l (low level) to 25 mg/l (high level), because of the increase in the ratio of pollutant to adsorbent, the removal rate increased from 73 to 85%. However, at high concentration, because of the saturation of adsorbent pores, the removal efficiency decreases. Dehghani et al. studied the efficiency of Fenton advanced oxidation process in the removal of sulfadiazine antibiotic from the aqueous solution at the initial antibiotic concentrations of 0.079, 0.19, 0.37 M by the batch experiments. Their results showed that adsorption rate decreased with an increase in initial concentration (Dehghani et al. 2012). Their results were consistent with our results for increasing of initial concentration from 25 to 50 mg/l. pH is another factor had a main effect on the adsorption process. An increase in pH from 4 to 7, acidic pH, (level 1 to level 2) resulted in an increase in removal efficiency from 70 to 87%, and by a further increase of the pH to 9, alkaline pH, (level 3), removal efficiency decreased to 78%. The obtained result was consistent with the result of the study conducted by Kakavandi et al. (2014) that the magnetized activated carbon powder was used to remove amoxicillin from aqueous solutions at pH 3 to 11. Their results showed that an increase in pH to 5, the removal efficiency increased and then the more increase of pH, removal efficiency decreased (Kakavandi et al. 2014).

According to the curves of the main effect on adsorption process, it is also possible to check out the role of adsorbent dose. Increasing the adsorbent dose from 0.3 to 0.5 mg (level 1 to level 2) resulted in an increase in adsorption efficiency from 77 to 82%, and by further increases in adsorbent dose to 0.7 mg (level 3), removal efficiency decreased 76%. A study conducted by Eslami et al. used 1 to 45 mg of the modified mesoporous silica (SBA-15) by modification with Trithiane as a new effective adsorbent for removing the mercury from aqueous solution. They found that the optimal dose of adsorbent was 15 mg and the removal efficiency fixed with increasing of adsorbent dose (Bidhendi et al. 2014). In Fig. 4, some lines are not parallel, and it represents some evidence for effective interactions between the parameters. It is clear that there is a significant interaction between pH and adsorbent dose on bisacodyl removal efficiency that an increase from low level to high level, this interaction increases and it is maximized at level 3.

The result of experiments indicated that the maximum efficiency of mesoporous silica in bisacodyl removal was obtained 88% at pH 7 (level 2), initial concentration of 50 mg/l (level 3), an adsorbent dose of 0.7 mg (level 3) and contact time of 24 h. While, the optimal conditions for removing of bisacodyl were suggested at pH = 7 (level 2), initial concentration of 50 mg/l (level 3), adsorbent dose of 0.3 mg (level 1) and contact time of 18 h, based on laboratory results, and the removal efficiency of bisacodyl was determined 78% at this condition.

The correlation coefficients of regression indicated that the adsorption isotherm is followed by Langmuir better than the Freundlich equation. The Langmuir isotherm assumes monolayer adsorption onto a surface with a finite number of specific sites. According to Langmuir isotherm, the monolayer saturation capacity of synthesized mesoporous silica was obtained 7.31 mg g^−1^ for bisacodyl. The Langmuir constant (b) is related to the affinity of the binding sites toward the adsorbate that was found to be 0.17. The Langmuir constant can be used for calculation of dimensionless separation factor, which indicate whether an adsorption process is ‘favorable’ or ‘unfavorable.’ This separation factor was 0.29 that predicts the adsorption is favorable at optimum condition.

The kinetic experimental data of bisacodyl adsorption process by mesoporous silica were well fited with pseudo-first order and pseudo-second order kinetic equation (R^2^ > 0.9). The pseudo-first kinetic indicates that the rate limiting step of the adsorption process may be chemical sorption involving electrical forces between adsorbent and adsorbate. The study conducted by Samadi et al. (2014), carbon nanotubes adsorption process for removing of antibiotics amoxicillin followed Langmuir isotherm and second order kinetic model (Samadi et al. 2014).

## Abbreviations

UV–Vis: ultra violet and visible
ANOVA: analysis of variance
DOE: design of experiments
CTAB: cetyl trimethyl ammonium bromide
TEOS: tetra-ethyl ortho silicate
SEM: scanning electron microscope
RPM: rate per minute
D.F: degree of freedom
